# Electronic structure and thermoelectric properties of Mo-based dichalcogenide monolayers locally and randomly modified by substitutional atoms†

**DOI:** 10.1039/d0ra08463h

**Published:** 2020-11-26

**Authors:** M. Vallinayagam, M. Posselt, S. Chandra

**Affiliations:** Helmholtz-Zentrum Dresden-Rossendorf, Institute of Ion Beam Physics and Materials Research Bautzner Landstraße 400 01328 Dresden Germany m.posselt@hzdr.de; Technische Universität Dresden 01062 Dresden Germany; Materials Science Group, Indira Gandhi Centre for Atomic Research, HBNI Kalpakkam 603102 Tamil Nadu India sharat.c@gmail.com

## Abstract

Density functional theory and Boltzmann transport equations are used to investigate electronic band structure and thermoelectric (TE) properties of different two-dimensional (2D) materials containing Mo, S, Nb, Se, and Te. In MoS_2_-based monolayers (MLs) the substitution of S atoms by Te atoms up to the concentration of 12.5 at% leads to a more significant change of the band structure than in the corresponding case with Se atoms. In particular, the bandgap is reduced. At a high concentration of Se or Te the electronic structure becomes more similar to that of the SeMoS or TeMoS Janus layers, and the MoSe_2_ or MoTe_2_ MLs. It is found that local and random introduction of substitutional Se or Te atoms yields not very different results. The substitution of Mo by Nb, at the concentration of 2.1 at% leads to hole levels. The thermoelectric properties of the considered 2D materials are quantified by the Seebeck coefficient and thermoelectric figure of merit. The two characteristics are determined for different levels of p- or n-doping of the MLs and for different temperatures. Compared to the pristine MoS_2_ ML, Te substitutional atoms cause more changes of the thermoelectric properties than Se atoms. However, MLs with Se substitutional atoms show a high thermoelectric figure of merit in a broader range of possible p- or n-doping levels. In most cases, the maximum thermoelectric figure of merit is about one, both in p- and n-type materials, and for temperatures between 300 and 1200 K. This is not only found for MoS_2_-based MLs with substitutional atoms but also for the Janus layers and for MoSe_2_ or MoTe_2_ MLs. Interestingly, for MLs with one Nb as well as two or four Te substitutional atoms the highest values of the TE figure of merit of 1.2 and 1.40, respectively, are obtained at a temperature of 1200 K.

## Abstract 2

Controlling electronic and thermoelectric properties of MoS_2_ monolayers by changing concentration of Se and Te chalcogenide.

## Graphical abstract

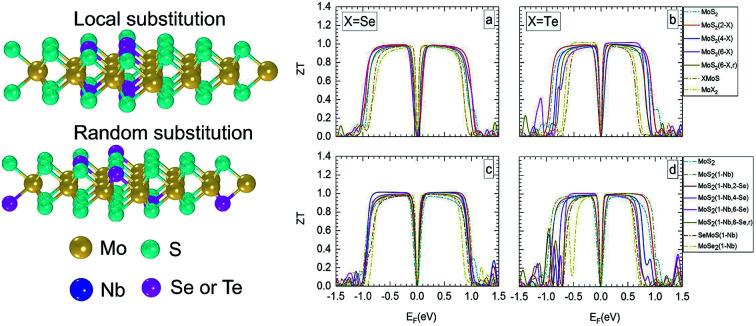

## I. Introduction

The direct conversion of heat into electricity is feasible using thermoelectric (TE) materials and creates huge interest in clean energy production. The efficiency of these materials depends mainly on Seebeck coefficient, electrical conductivity, and thermal conductivity.^1,2^ The TE efficiency can be improved in many aspects such as replacing bulk solids with materials with reduced dimension.^1–13^ In particular Transition Metal Dichalcogenides (TMD) attract much interest due to the control over the electronic properties *via* various mechanisms such as compositional modifications,^14–19^ layer stacking,^20,21^ applying external fields,^22,23^ layer interfacing,^24^ and inducing strain on layers.^23,25,26^ Depending on the composition TMD show various characteristics such as metallic, semiconducting and superconducting properties.^27–32^ Therefore, TMD may be interesting for many applications.^32–34^ TMD are layered bulk material and each layer consist of three atomic planes. Two-dimensional TMD consisting of a monolayers are synthesized from bulk TMD using exfoliation process or chemical treatment on suitable substrate.

Dimensionality reduction, from three to two dimension, influences bandgap and may cause indirect to direct bandgap transition.^2,6,13,20,35,36^ Tuning the electronic properties, which influence carrier concentration and mobility as well as Seebeck coefficient and thermal conductivity, is vital for the TE applications of TMD. Recent theoretical^16^ and experimental^18,37,38^ investigations on monolayer (ML) MoS_2_ with substitutional Se and Te atoms show that the electronic band gap changes due to S–Se and S–Te interactions. The band gap values are in the range of 1.0–1.8 eV.^39^ On the other hand, Janus MLs have been formed where one of the atomic plane consisting of S is replaced by a plane with other chalcogenides, *e.g.* Se or Te. Experimental^40^ and theoretical studies^41^ were performed to investigate the TE properties of Janus layers XMoX′ (X′ and X may be S, Se, or Te, with X′ ≠ X). Calculations show that Janus MLs XMoX′ but also SePtS have a higher TE performance under applied strain.^42,43^

Many studies investigated properties of TMD MLs by the introduction of substitutional atoms at different concentrations.^16,18,37,39,44^ For example, Komsa *et al.*^16^ theoretically studied MoS_2_ MLs with different concentrations of random substitution of S by Se or Te. They showed that MoS_2(1−*x*)_Se_2*x*_ and MoS_2(1−*x*)_Te_2*x*_ MLs are thermodynamically stable and the bandgap changes when Se/Te concentration is varied.^16^ On the other hand, Feng *et al.*^15,37^ succeeded in synthesizing MoS_2(1−*x*)_Se_2*x*_ MLs. They measured the bandgap using photoluminescence spectroscopy.^15,37^ Using High-Resolution Transmission Electron Microscopy (HRTEM) and Atomic Resolution High-Angle Annular Dark-Field Scanning Transmission Electron Microscopy (HAADF-STEM) they found clusters with different MoS_2(1−*x*)_Se_2*x*_ compositions embedded in the MoS_2_ ML.^15,37^

In the present work band structure and TE properties of the pristine MoS_2_ ML are compared with those of MoS_2_-based MLs modified by random or local substitution of S by Se or Te. Local substitution means that some or all S atoms which are nearest neighbors of a certain Mo atom are replaced. Obviously, in a real sample, both the random and cluster arrangement of substitutional atoms may exist, and until now it is not clear how the different positions of Se or Te influence band structure and TE properties. Additionally, the substitution of Mo by Nb is considered. Moreover, electronic structure and TE characteristics of SeMoS and TeMoS Janus layers as well as MoSe_2_ and MoTe_2_ MLs, with or without Nb substitutional atom are determined. In this manner, the properties of Mo-based dichalcogenide MLs are determined for low to maximum concentration of S substitution by Se or Te, for cases without or with Nb substitution of Mo. To the best of our knowledge, such a comprehensive study has not been performed yet. The results obtained for the different MLs are compared in order to find optimum TE features.

## II. Structural models and computational details

**Fig. 1:**
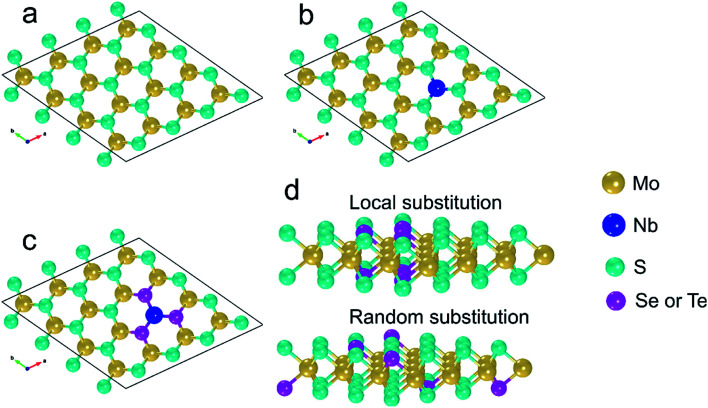
View onto the *a*–*b* plane: (a) pure MoS_2_ ML, (b) MoS_2_(1-Nb), (c) MoS_2_(1-Nb,6-X). (d) Cross sectional view of and comparison between local and random substitution of X. Here X = Se, Te.

The models of pure and compositionally modified MLs are derived from the bulk 2H-MoS_2_ polymorph which shows *P*6_3_/*mmc* symmetry.^32^ The bulk unit cell has two Mo and four S atoms, which are arranged alternately in 3 planes (2 S planes and one Mo plane in between). Each Mo atom has six nearest S and each S has three nearest Mo atoms, all together forming trigonal prismatic coordination.^32^ In this work, a supercell based on the pristine MoS_2_ ML is considered (Fig. 1a). In this supercell, the basic MoS_2_ ML consists of 4 × 4 MoS_2_ unit cells, *i.e.* of 16 Mo and 32 S sites. Three-dimensional periodic boundary conditions are applied. Along the *c* axis, the distance between the periodic replicas of the MLs is 15 Å so that interlayer interactions are avoided. The following locally modified MoS_2_-based MLs are investigated: (i) in the pure MoS_2_ ML one Mo atom is replaced by one Nb atom. This is denoted by MoS_2_(1-Nb) (Fig. 1b). One Nb atom in the given supercell corresponds to an atomic concentration of about 2.1 at%, due to cell size and periodic boundary conditions. Nb is energetically stable when it occupies Mo sites as demonstrated experimentally by Wu *et al.*^45^ (ii) The six S atoms surrounding the substitutional Nb atom are replaced one by one by Se atoms. Here the denotations MoS_2_(1-Nb,1-Se), …, MoS_2_(1-Nb,6-Se), or shortly MoS_2_(1-Nb,1–6-Se), are used. This corresponds to Se concentrations between 2.1 and 12.5 at%. An example is shown in Fig. 1c. (iii) Similarly to Se incorporation, Te atoms are substituted on S sites, which is denoted by MoS_2_(1-Nb,1–6-Te). Also cases without the Nb substitutional atom, *i.e.* (iv) MoS_2_(1–6Se) and (v) MoS_2_(1–6Te), are studied to determine the effect of Nb. In order to investigate the difference between local and random modification of MoS_2_ MLs we consider random substitution of 6 S atoms by 6 Se or 6 Te atoms with the denotations MoS_2_(6-Se,r), MoS_2_(6-Te,r), MoS_2_(1-Nb,6-Se,r) and MoS_2_(1-Nb,6-Te,r), see Fig. 1d. Furthermore, the following Janus MLs are investigated: (i) the S atoms in one atomic plane of the pristine MoS_2_ ML are replaced by Se atoms, (ii) or by Te atoms, (iii) in the Janus ML of case (i) one Mo is replaced by Nb, and (iv) in the Janus ML of case (ii) one Mo is replaced by Nb. The following denotations are used for the Janus MLs (i) SeMoS, (ii) TeMoS, (iii) SeMoS(1-Nb), and (iv) TeMoS(1-Nb). Also MoSe_2_ and MoTe_2_ MLs are considered in this work. The respective supercells are constructed from the corresponding bulk crystal using the same procedure as for the MoS_2_ ML. Pristine as well as MLs with one Nb atom, *i.e.* MoSe_2_(1-Nb) and MoTe_2_(1-Nb), are investigated. The ball-stick representations shown in Fig. 1 were generated using VESTA software.^46^

First principle calculations are performed using the VASP code,^47^ with projector augmented wave (PAW) pseudopotentials^48^ and the local density approximation (LDA) to treat exchange and correlation effects. The plane-wave energy cutoff is set to 400 eV. The atomic positions are relaxed and optimized until the Hellmann–Feynman forces on all individual atoms become lower than 10^−5^ eV Å^−1^ and total energy values converged up to 10^−9^ eV. Due to the choice of the given supercell for the MLs, a Monkhorst–Pack^49^ grid of 5 × 5 × 1 *k*-points is used for Brillouin zone sampling. To achieve a high accuracy in the calculation of the density of electronic states (DOS) a denser *k*-point grid of 24 × 24 × 1 *k*-points is used, and the tetrahedron method with Blöchl corrections^50^ is adopted.

Before investigating the different kinds of MLs, DFT calculation for bulk 2H-MoS_2_ is performed where the atomic positions as well as the volume and shape of the simulation cell are relaxed in order to obtain the correct ground state configuration. In this case, a supercell corresponding to the bulk 2H-MoS_2_ unit cell and a 12 × 12 × 12 *k*-point grid is employed. The supercell with the ML (see above) is then built using the atomic distances obtained for bulk 2H-MoS_2_. In investigations of the different MoS_2_-based MLs with substitutional atoms, the size and shape of the supercell are kept constant but the positions of atoms are relaxed. Also for the Janus layers SeMoS, TeMoS, SeMoS(1-Nb), and TeMoS(1-Nb) the same supercell size and shape as for the pristine MoS_2_ ML is used and only the ionic positions were relaxed.

The DFT results are used as inputs for subsequent thermoelectric calculations with the code BoltzTraP.^51^ Results are the Seebeck coefficient *S*, the electrical conductivity *σ*, and the electronic part of thermal conductivity *κ*_e_. The Seebeck coefficient *S* describes the voltage built up in the material if a temperature gradient is applied, and is inversely related to carrier concentration and directly related to temperature and carrier effective mass.^1^ High effective mass may result in high *S* values but leads to low carrier mobility and low electrical conductivity. Hence, all relevant quantities are combined to determine the TE figure of merit, which characterizes quality of TE materials,1
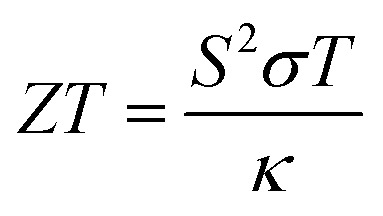
where *T* is the absolute temperature, and *κ* is the thermal conductivity, which is sum of contributions from electronic (*κ*_e_) and phonon (*κ*_ph_) parts. In the present work, the thermoelectric properties are determined for temperatures of 300, 500, 800, and 1200 K.

## III. Results and discussion

### A. Electronic band structures

Using VASP, lattice parameters and band gaps of bulk MoS_2_ and of the pristine MoS_2_ ML were calculated. In Table 1 these results are compared with previous theoretical and experimental data. Our values for bulk MoS_2_ deviate by less than 2% from experimental data and closely agree with previous theoretical reports. In particular, the *c*/*a* ratio (3.864) is rather similar to the experimental value of 3.892 obtained from Angle-Resolved Photoemission Spectroscopy (ARPES).^52^ The calculated band gap of bulk MoS_2_ closely matches with theoretical values of ref. 21 and 53. Our calculated band gap for MoS_2_ ML agrees well with theoretical data reported in ref. 27, 37 and 53 and also with experimental value.^37,53^

**Table 1:** Lattice parameters and band gap of bulk and ML MoS_2_: comparison with available theoretical and experimental data

	MoS_2_ bulk				MoS_2_ ML			
	This work	Literature	Code	Remark	This work	Literature	Code	Remark
*a* (Å)	3.123	3.169 (ref. 21)	VASP	GGA	3.123	3.180 (ref. 56)	VASP	GGA
		3.130 (ref. 53)	VASP	LDA		3.125 (ref. 55)	ABINIT	LDA
		3.127 (ref. 55)	ABINIT	LDA		3.118 (ref. 35)	PWSCF	LDA
		3.150 (ref. 54)	AIMPRO	LDA				
		3.160 (ref. 28)	Exp					
*c* (Å)	12.068	12.324 (ref. 21)	VASP	GGA	—	—	—	—
		12.040 (ref. 53)	VASP	LDA				
		12.066 (ref. 55)	ABINIT	LDA				
		12.290 (ref. 54)	AIMPRO	LDA				
		12.294 (ref. 28)	Exp	Exp				
*E*_g_ (eV)	0.750	0.890 (ref. 21)	VASP	GGA	1.85	1.870 (ref. 27)	VASP	LDA
		0.710 (ref. 53)	VASP	LDA		1.670 (ref. 56)	VASP	GGA
		1.170 (ref. 54)	AIMPRO	LDA		1.860 (ref. 37)	Exp	
		1.23 (ref. 57)	Exp			1.840 (ref. 53)	Exp	

After substituting one Mo by one Nb in the MoS_2_ ML, Nb–S bond length of 2.429 Å is obtained which is in agreement with the theoretical value 2.500 Å reported by Ivanovskaya *et al.*^54^ This clearly shows that Nb substitution meagerly affects the local atomic structure. In MoS_2_(1-Nb,6-Se) and in MoS_2_(1-Nb,6-Te) the Nb–Se and Nb–Te bond lengths are calculated to be 2.542 and 2.733 Å, respectively, which is very similar to the theoretical values reported by Ataca *et al.*^27^ Furthermore, for MoS_2_(6-Se) and MoS_2_(6-Te) Mo–Se and Mo–Te bond lengths of 2.494 and 2.679 Å were obtained, respectively.

The bandgap data of all the MLs considered in this work are summarized in Table 2. If the S atoms near a certain Mo atom are gradually substituted by Se, the bandgap is slightly reduced compared to pristine MoS_2_. The trend of the computed bandgap for MoS_2_(*n*-Se), *n* = 1, …, 6, matches with experimental data for MoS_2(1−*x*)_Se_2*x*_ samples prepared by chemical^37,61^ vapor deposition. The bandgap of MoS_2_(6-Se,r) and MoS_2_(6-Te,r) layers is somewhat higher than that for local substitutions of S by Se or Te. A reduction of the bandgap is found if one (SeMoS) or two (MoSe_2_) S layers are completely replaced by Se layers. In these cases, and also for MoS_2_(6-Se) and MoS_2_(6-Se,r), as well as for SeMoS and MoSe_2_, an indirect bandgap is found. Replacing the Mo atom close to the gradually introduced Se atoms by Nb leads to significant changes compared to the case without this replacement. The bandgap of MoS_2_(1-Nb,6-Se,r) is somewhat larger than that of the MoS_2_(6-Se,r), whereas the opposite is found in the case of Te. Substituting one Mo by Nb in MoS_2_(6-Se), MoS_2_(6-Se,r), SeMoS, and MoSe_2_ turns the indirect bandgap to a direct one. The bandgap value and type found for MoSe_2_ closely matches with previous theoretical results^27^ whereas the theoretical bandgap of SeMoS found in ref. 59 is smaller than the value obtained in this work.

**Table 2:** The band gap of MoS_2_(*n*-X), MoS_2_(1-Nb,*n*-X), Janus and MoX_2_ MLs (*n* = 1–6; X = Se, and Te). The bandgaps marked with an asterisk are indirect. Experimental and theoretical bandgap data from literature are given in bold face and italics, respectively

	*E*_g_ (eV)			*E*_g_ (eV)	
	X = Se	X = Te		X = Se	X = Te
MoS_2_	1.85		MoS_2_(1-Nb)	1.83	
	(*1.87* (ref. 27))			(*1.75* (ref. 58))	
	**(1.86 (ref. 37))**			**(1.80 (ref. 17))**	
MoS_2_(1-X)	1.83	1.77	MoS_2_(1-Nb,1-X)	1.82	1.76
MoS_2_(2-X)	1.85	1.78	MoS_2_(1-Nb,2-X)	1.80	1.62
MoS_2_(3-X)	1.78	1.65	MoS_2_(1-Nb,3-X)	1.79	1.49
MoS_2_(4-X)	1.78	1.58	MoS_2_(1-Nb,4-X)	1.77	1.32
MoS_2_(5-X)	1.79	1.52	MoS_2_(1-Nb,5-X)	1.75	1.31
MoS_2_(6-X)	1.72*	1.49	MoS_2_(1-Nb,6-X)	1.73	1.33
MoS_2_(6-X,r)	1.84*	1.53	MoS_2_(1-Nb,6-X,r)	1.90	1.45
XMoS Janus	1.65*	1.01*	XMoS(1-Nb) Janus	1.67	1.09*
	(*1.55* (ref. 59))	(*1.00* (ref. 59))			
MoX_2_	1.61*	1.24	MoX_2_(1-Nb)	1.56	1.20
	(*1.62* (ref. 27))	(*1.25* (ref. 27))			
	**(1.55 (ref. 37))**	**(1.10 (ref. 60))**			

The gradual replacement of S near a certain Mo by one to six Te atoms yields a stronger reduction of the bandgap than substitution by Se. If the Mo atom near the Te atoms is substituted by Nb this reduction is still more pronounced. The smallest value of *E*_g_ is found for TeMoS and TeMoS(1-Nb), and the corresponding bandgap is indirect. A good agreement with literature data^27,59^ is found for the bandgap values of TeMoS and MoTe_2_. MoTe_2_ and MoTe_2_(1-Nb) exhibit a direct bandgap which is slightly wider than that of the Janus layers. The difference between bandgaps of MoS_2_ and MoTe_2_ as well as between MoS_2_ and MoSe_2_ (*E*^MoS^_g_^_2_^ − *E*^MoSe^_g_^_2_^ = 0.24 eV and *E*^MoS^_g_^_2_^ − *E*^MoTe^_g_^_2_^ = 0.61 eV) explains why the bandgap decreases more in MoS_2_(1–6Te) and MoS_2_(1-Nb,1–6Te) than in MoS_2_(1–6Se) and MoS_2_(1-Nb,1–6Se).

**Fig. 2:**
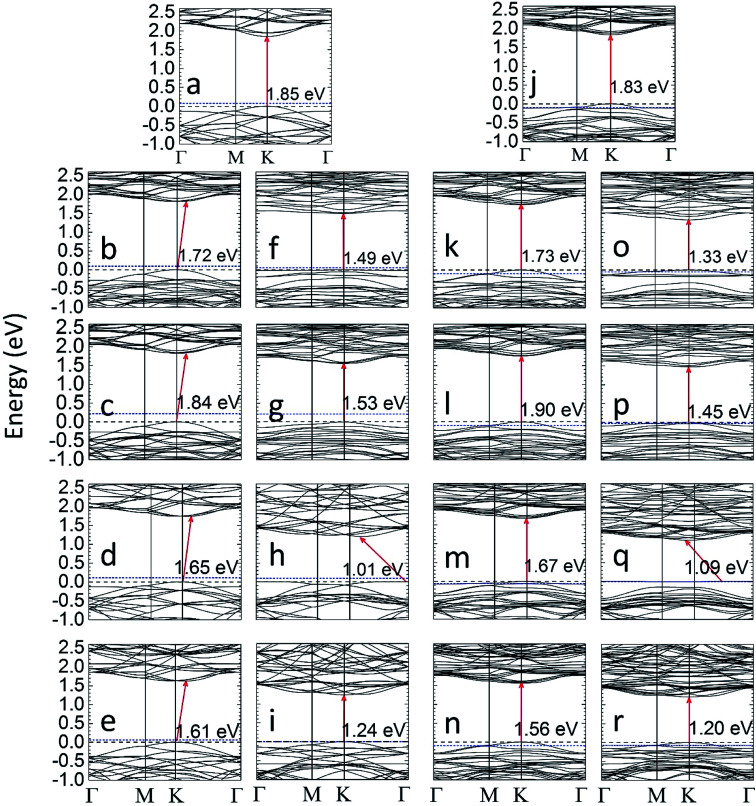
The calculated band structure of MLs without (a–i) and with (j–r) a Nb substitutional atom. (a) MoS_2_, (b) MoS_2_(6-Se), (c) MoS_2_(6-Se,r), (d) SeMoS Janus layer, (e) MoSe_2_, (f) MoS_2_(6-Te), (g) MoS_2_(6-Te,r), (h) TeMoS Janus layer (i), MoTe_2_, (j) MoS_2_(1-Nb), (k) MoS_2_(1-Nb,6-Se), (l) MoS_2_(1-Nb,6-Se,r), (m) SeMoS(1-Nb) Janus layer, (n) MoSe_2_(1-Nb), (o) MoS_2_(1-Nb,6-Te), (p) MoS_2_(1-Nb,6-Te,r), (q) TeMoS(1-Nb) Janus layer, (r) MoTe_2_(1-Nb). The valence band maximum (VBM, dashed black line) is set to 0 eV and the Fermi level (blue dotted line) is given with respect to the VBM.

Selected results of band structure calculations are depicted in Fig. 2. The energy scale is chosen in such a manner that in all cases the valence band maximum (VBM) is set at 0 eV and the Fermi energy determined by VASP is set with respect to VBM or 0 eV. The substitution of one Mo by one Nb may lead to hole levels (see Fig. 2j–r), since Nb is a single acceptor. Such local levels turn MoS_2_(1-Nb), MoS_2_(1-Nb,6-X), and MoS_2_(1-Nb,6-X,r) layers to p-type materials, which is in agreement with experimental observation^17^ and theoretical investigation.^58^ The similar effect is observed for SeMoS(1-Nb), MoSe_2_(1-Nb) and MoTe_2_(1-Nb). Both the local and random substitution of Se leads to similar VBM and CBM shapes. A peculiar effect is found in Fig. 2f, o and q: there are flat levels at the top of the valence band. This could indicate that these electronic states are not hybridize with the others. This is obviously caused by the presence of Se or Te, which also leads to a reduction of the band gap. Furthermore, the total and partial density of states (DOS) of the differently modified MLs were calculated. The results are shown in Section SA (Fig. S1 and S2) of the ESI.†^62^ The presence of Se or Te in MoS_2_(1–6Se) and MoS_2_(1–6Te) MLs leads to a gradual modification of the Mo(d) and S(p) orbitals which is more pronounced in the valence band. Te substitutional atoms lead to a larger localization of electrons near the valence band maximum than Se. A Nb substitutional atom generates hole levels which was already discussed in connection with Fig. 2. The DOS results show that these levels are more localized in cases with substitutional Te than in those with substitutional Se. Obviously, the interplay between Nb(d) orbitals with Se(p) and Te(p) orbitals is different. The DOS of the Janus layers and of MoSe_2_ as well as MoTe_2_ MLs, without or with a Nb atom, shows trends regarding the influence of Se, Te, and Nb similar to those found for the locally modified MoS_2_ MLs.

Furthermore, the formation or substitution energy of Nb, Se, and Te in MoS_2_ was determined. Details of the calculations can be found in Section SB of the ESI.†^62^ The energy for Nb substitution at Mo site is −4.720 eV (S-rich condition) and −0.006 eV (Mo-rich condition), which clearly shows the feasibility of Nb incorporation under S-rich conditions (see Table S2 in ESI†^62^). Our values deviate from the those reported in ref. 58 by 1.57 eV (S-rich condition) and 0.18 eV (Mo-rich condition). This is certainly due to different simulation settings. However, our results qualitatively agree well with those of ref. 58, showing that S-rich conditions are more favorable than Mo-rich condition to obtain a p-type MoS_2_-based ML by Nb incorporation. Similar results are reported by Ivanovskaya *et al.*^54^ In the case of local or random substitution of S the substitution energy of Se or Te is negative (positive) under Mo-rich (S-rich) condition, see Tables S1 and S2 of the ESI.†^62^ This suggests that practical preparation of MLs with substitutional atoms Se or Te is energetically feasible under Mo rich condition. The formation energy data show that a simultaneous incorporation of Nb and Se (or Te) is difficult, because opposite chemical environments are required for sufficiently stable Nb and Se (or Te) substitutional atoms. Replacement of a whole S layer by Se or Te is also favorable under Mo rich conditions, see Tables S1 and S2 of the ESI.†^62^ The results presented in the ESI† clearly show that irrespective of Se or Te concentration levels, S substitution by Se is more favorable than substitution by Te. This fact should be due to larger ionic size of Te (ionic radius of 2.10 Å) than that of Se (ionic radius of 1.90 Å).

### B. Thermoelectric properties

#### 1. Seebeck coefficient

**Fig. 3:**
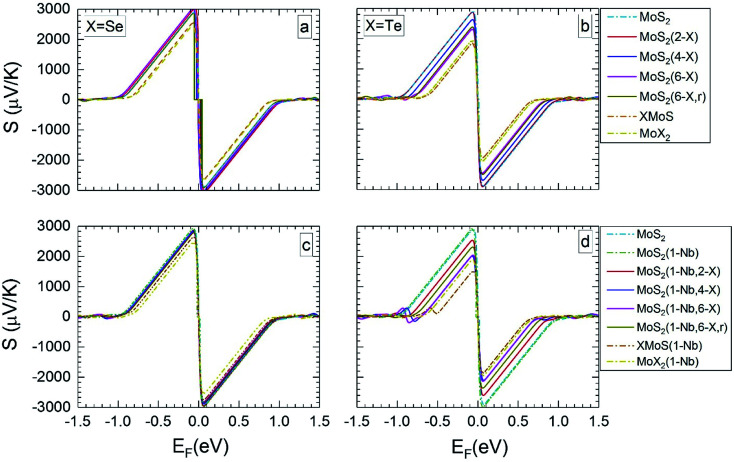
Seebeck coefficients at 300 K *versus* the Fermi level, for the different MLs, with X = Se or Te.

**Fig. 4:**
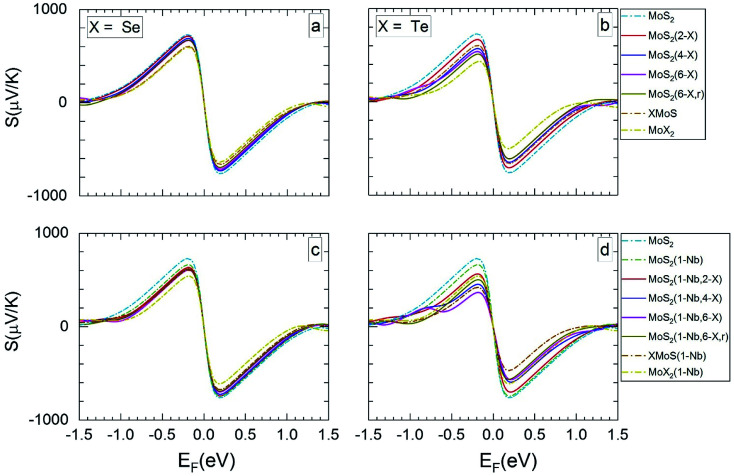
The calculated Seebeck coefficients at 1200 K *vs.* Fermi level, for the same MLs as presented in Fig. 3.

Fig. 3 and 4 present the dependence of the Seebeck coefficient *S* on the Fermi level *E*_F_ for temperatures of 300 and 1200 K, respectively. Further data for 500 and 800 K are given in Section SC of the ESI (Fig. S4 and S5).†^62^ Following the standard representation in literature, here the Fermi energy is set to zero at the mid bandgap. Hence, the negative and positive values of Fermi levels indicate p- and n-doping cases. In Fig. 3 and 4 as well as in Fig. S4 and S5† the average value of Seebeck coefficient is given, which is obtained from data of in-plane and out-of plane Seebeck coefficients. At 300 K the Seebeck coefficient shows a larger variation with Fermi level than at 1200 K. The maximum (and minimum) of the Seebeck coefficient *S*_max_ (*S*_min_) of the pristine MoS_2_ ML and of MLs with Se substitutional atoms (Fig. 3a and 4a) are similar, since the corresponding band structures are not very different. However, *S*_max_ (and *S*_min_) of the Janus layer SeMoS and of the MoSe_2_ ML is somewhat lower due to the smaller band gap. The effect of Nb substitution on *S* is more pronounced when the temperature is increased. At room temperature both pristine and Nb substituted MLs give almost same *S* values. At 1200 K *S* of the pristine MoS_2_ ML is somewhat higher than that with the Nb substitutional atom.

For MLs with locally positioned Te atoms without (Fig. 3b and 4b) and with (Fig. 3d and 4d) Nb *S*_max_ (and *S*_min_) decreases with increasing Te content. The lowest value of *S*_max_ (and *S*_min_) is obtained for the TeMoS Janus layer and/or for MoTe_2_. The reduction with increasing Te content is still somewhat larger in case with the Nb. Reduction of *S*_max_ (and *S*_min_) may be explained by the decrease of the bandgap (see Table 2). At 300 K the variation of the Seebeck coefficient of MoS_2_(1-Nb,6-Te,r) is slightly higher than MoS_2_(1-Nb,6-Te). This difference is more pronounced at 1200 K.

A direct comparison with literature data of the Seebeck coefficient is difficult. Hong *et al.*^22^ showed data of in-plane Seebeck data of the pristine MoS_2_ ML at 300 K. For a proper comparison, their energy scale was adopted to the band gap determined in the present work, and *E*_F_ was set to zero at midgap (see above). A good agreement with our data is found. For example, our value of 1200 μV K^−1^ at *E*_F_ = −0.60 eV (Fig. 3a) matches with 1200 μV K^−1^ obtained in ref. 22 at the corresponding Fermi level. A similar adoption or rescaling was used to compare with the in-plane Seebeck coefficient of Wickramaratne *et al.*^36^ for pristine MoS_2_ ML at 300 K. Also in this case our values match well with the literature data, *e.g.* at *E*_F_ = −0.63 eV (Fig. 3a) with *S* = 1000 μV K^−1^.

#### 2. Thermoelectric figure of merit

The efficiency of a thermoelectric device is characterized by the TE figure of merit *ZT*, see eqn (1). The Seebeck coefficient *S*, the electrical conductivity *σ* as well as the electronic part of the thermal conductivity *κ*_e_ are determined by the BoltzTraP code, whereas the phonon contribution to the thermal conductivity *κ*_ph_ is obtained using the following considerations. The results presented in Section III.A indicate that the band structure of MLs with Se substitutional atoms is not very different to that of the pure MoS_2_ ML. In this case we assume that the phonon contribution *κ*_ph_ to the thermal conductivity is the same as that of pristine MoS_2_ ML and use the data from ref. 63. For MLs with Te substitutional atoms and the TeMoS Janus layer, the band structure shows a larger deviation from that of pure MoS_2_ ML, indicating a strong influence of Te. Therefore, we employ the phononic thermal conductivity data for the pristine MoTe_2_ ML from ref. 64.

**Fig. 5:**
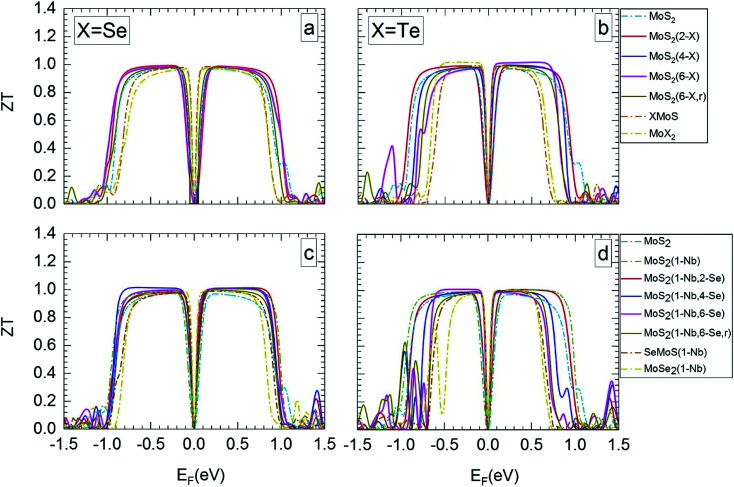
Thermoelectric figure of merit at 300 K in dependence on Fermi level, for the different MLs, with X = Se or Te.

**Fig. 6:**
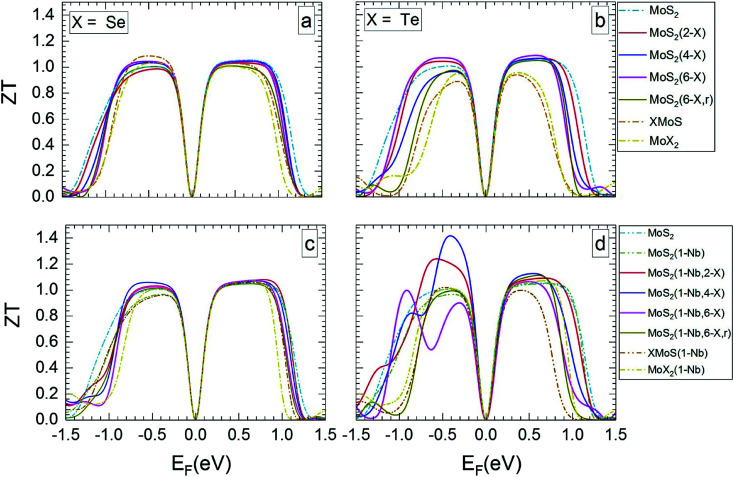
Thermoelectric figure of merit at 1200 K in dependence on Fermi level, for the MLs presented in Fig. 5.

Fig. 5 and 6 show *ZT* for the different MLs *versus* the Fermi level for temperatures of 300 and 1200 K, respectively. *ZT* results for 500 and 800 K can be found in Section SD of the ESI.†^62^ Like the Seebeck coefficient (Fig. 3 and 4) the TE figure of merit shows a significant dependence on the electrical doping, *i.e.* the Fermi level. In most cases, MoS_2_-based MLs modified by substitutional atoms show high *ZT* values of about 1 over a broader range of possible Fermi levels than Janus, MoSe_2_, and MoTe_2_ MLs.

MLs with Se substitutional atoms (Fig. 5a and c) exhibit high *ZT* values in a wider region of Fermi-level values than in the case of Te substitutional atoms (Fig. 5b and d). Optimum *ZT* values are found for the Se substituted MLs with a Nb substitutional atom (Fig. 5c). In this case *ZT* is nearly constant in most of the possible n- or p-regions, with the exception of those close to mid gap and at band edges. In comparison with Fig. 5a, the Fermi-level range of high *ZT* value is slightly extended due to the Nb substitutional atom. Here, various p-doping levels may be possible between about −0.7 and −0.1 eV, or various n-doping levels between about 0.1 and 0.8 eV, with a value of the TE figure of merit close to 1. There is only a small difference between *ZT* data for random and local substitution of S by Se or Te. In some cases random substitution leads to a slightly smaller range of Fermi levels with high *ZT* values. The results depicted in Fig. 5a–d clearly show that SeMoS or TeMoS Janus layers as well as the MoSe_2_ and MoTe_2_ MLs do not perform better than the pristine MoS_2_ ML at 300 K. Replacing Mo by Nb locally leads to minor improvement of *ZT* of the Janus layers.

Our maximum value of *ZT* of about 1 for the pristine MoS_2_ ML at 300 K is in relatively good agreement with literature data of 0.97(1.35) for the p(n) region.^36^ Others authors obtained data that differ considerably: 0.47(0.22),^56^ 0.51(0.23),^65^ and 0.25(0.68).^13^ This may be due to the different calculation schemes used in these publications.

The value of *ZT* slightly increases with temperature. At 1200 K (Fig. 6) the range of Fermi energies belonging to high *ZT* is somewhat narrower than at 300 K. In the case of MLs with substitutional Se atoms, as well as for the SeMoS Janus layer and the MoSe_2_ ML the temperature dependence of the main features is not very pronounced, as comparison of Fig. 5a and c with Fig. 6a and c shows. If the MLs contain Te substitutional atoms as well as in the case of the TeMoS Janus layer and the MoTe_2_ ML multiple peaks are found both at 300 and 1200 K. These features are also found at 500 and 800 K, see ESI,^62^ Fig. S6 and S7.† Multiple conduction channels due to multi-fold degenerate bands may explain the various peaks. Interestingly, MoS_2_(1-Nb,2-Te) and MoS_2_(1-Nb,4-Te) ML have the highest *ZT* value of 1.2 and 1.40, respectively at Fermi level of about −0.50 eV.

## IV. Conclusions

Density functional theory and calculations using Boltzmann transport equations were employed to determine the electronic structure and the thermoelectric properties of various two-dimensional materials containing Mo, S, Nb, Se, and Te. In the MoS_2_-based MLs the substitution of S atoms by Te atoms (concentrations between 2.1 and 12.5 at%) leads to a stronger modification of the band structure than in the corresponding case with Se atoms. With increasing concentration of Se or Te the electronic structure becomes similar to that of the SeMoS or TeMoS Janus layers, and also similar to that of the MoSe_2_ or MoTe_2_ MLs. It was found that local and random introduction of substitutional Se or Te atoms yields not very different results. The substitution of Mo by Nb (concentration of 2.1 at%) does not alter the band structure significantly but leads to hole levels near the valence band maximum.

The band structure data were used to calculate thermoelectric properties, in particular the Seebeck coefficient and the thermoelectric figure of merit. The two quantities were determined for different levels of p- or n-doping of the considered MLs and for different temperatures. The stronger influence of Te substitutional atoms on the band structure of MoS_2_-based MLs causes also more significant changes of the thermoelectric properties, compared to the pristine MoS_2_ ML. On the other hand, MLs with Se substitutional atoms show a high thermoelectric figure of merit in a broader range of possible p- or n-doping levels. In most cases investigated, the maximum thermoelectric figure of merit has a value of about one, both in p- and n-regions. This holds for temperatures between 300 and 1200 K, and not only for MoS_2_-based MLs with substitutional atoms but also for the Janus layers and for MoSe_2_ or MoTe_2_ MLs.

The comprehensive study performed in this work led to important data on properties of MoS_2_-based dichalcogenide MLs which had not yet been available. These results are very valuable for further experimental investigations as well as for the development and improvement of 2D materials.

## Conflicts of interest

There are no conflicts to declare.

## Supplementary Material

RA-010-D0RA08463H-s001
