# Supplementary material for: Electronic structure and thermoelectric properties of Mo-based dichalcogenide monolayers locally and randomly modified by substitutional atoms
Source: RSC Adv. 2020 Nov 26;10(70):43035–44. doi: 10.1039/d0ra08463h (PMC9058219; doi:10.1039/d0ra08463h)
Supplement: RA-010-D0RA08463H-s001 [file RA-010-D0RA08463H-s001.pdf]

Electronic Supplementary Material (ESI) for RSC Advances.
This journal is © The Royal Society of Chemistry 2020

Supplement material for

Electronic structure and thermoelectric properties of Mo-based dichalcogenide monolayers locally and randomly modified by substitutional atoms

M. Vallinayagam[1,2], M. Posselt[1§], S. Chandra[3*]

[1] Helmholtz-Zentrum Dresden-Rossendorf, Institute of Ion Beam Physics and Materials Research, Bautzner Landstraße 400, 01328 Dresden, Germany

[2] Technische Universität Dresden, 01062 Dresden, Germany

[3] Materials Science Group, Indira Gandhi Centre for Atomic Research, HBNI, Kalpakkam 603102, Tamil Nadu, India

*** sharat@igcar.gov.in**

§ m.posselt@hzdr.de

SA. Density of states

The effect of Se, Te, and Nb on the electronic density of states (DOS) is summarized in Figs. S1 and S2. In pristine MoS_2 ML Mo(d) orbitals contribute to both valence and conduction band region, see the energy intervals -2 to 0 eV and 2 to 3 eV. In the same energy intervals contribution of S(p) orbitals is observed. Mo(d) dominates in conduction band region. In the upper and lower part of the valence band Mo(d) and S(p) dominate, respectively. The presence of Se or Te in MoS_2(1-6Se) and MoS_2(1-6Te) MLs leads to a gradual modification of the Mo(d) and S(p) orbitals which is more pronounced in the valence band. A Nb substitutional atom generates hole levels in the case of MoS_2(1-Nb) (Fig. S2) but the behavior of Mo(d) and S(p) is rather similar to that for pristine MoS_2. Hole levels are also observed in MoS_2(1-Nb,1-6Se), and MoS_2(1-Nb,1-6Te). Similar results were obtained in Refs. [1, 2]. The DOS results show that these levels are more localized in cases with substitutional Te than in those with substitutional Se. Obviously, the interplay between Nb(d) orbitals with Se(p) and Te(p) orbitals is different.

In the DOS of the SeMoS Janus layer (Fig. S1) both S(p) and Se (p) contributions can be seen in valence band region and almost overlap with each other except for peak intensities. As seen in ML MoS_2, Mo (d) occupies valence and conduction band regions, but dominates in the latter. As in case of MoS_2 MLs with substitutional Se or Te atoms the presence of Nb leads to hole levels. The levels in SeMoS(1-Nb), and TeMoS(1-Nb) layers originate from different contributions. In case of SeMoS(1-Nb), the impurity levels are due to hybridation between Se(p)-S(p)-Nb(d)-Mo(d), see the inset in SeMoS(1-Nb) DOS.

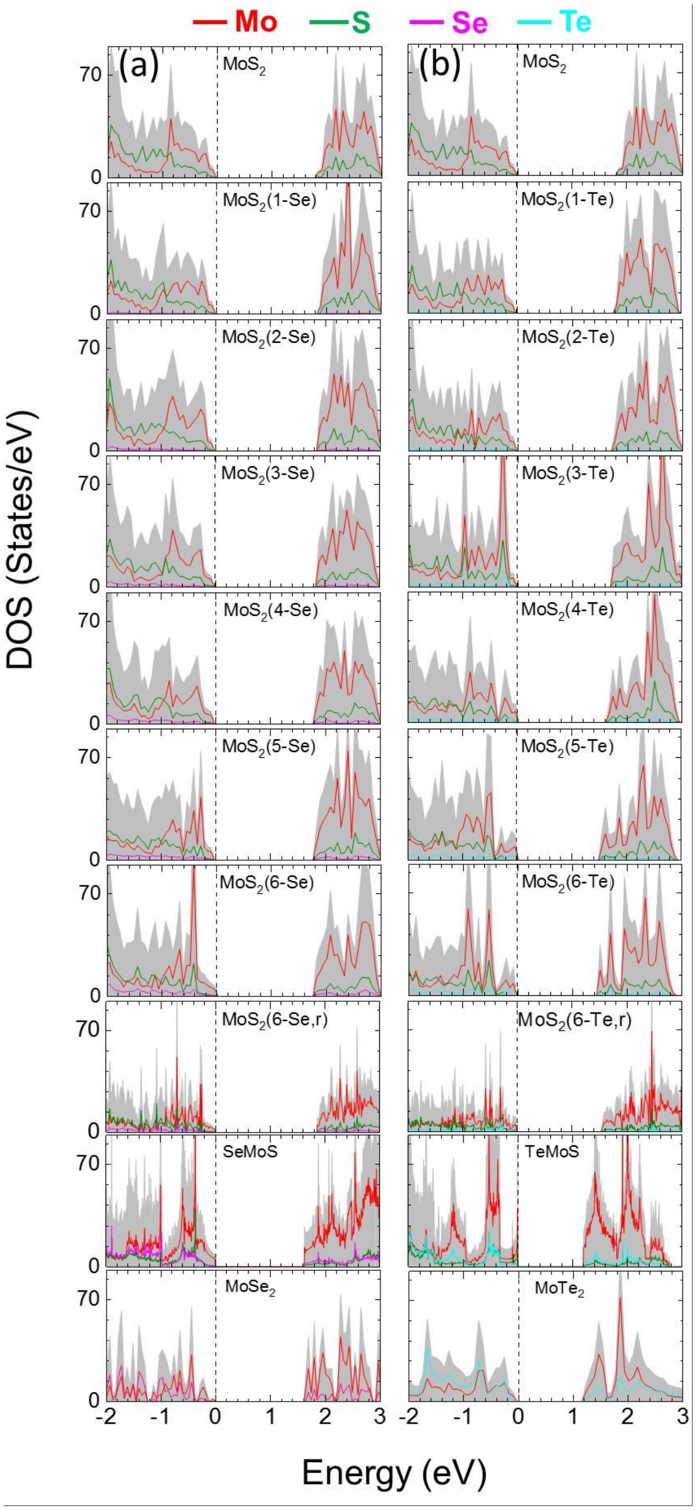

FIG. S1: Total and partial density of states of MoS_2, MoS_2(n-Se), SeMoS, $MoSe_2$ (left column) and MoS_2(n-Te), TeMoS, $MoTe_2$ (right column). Here n=1-6 and the valence band maximum was set to 0 eV.

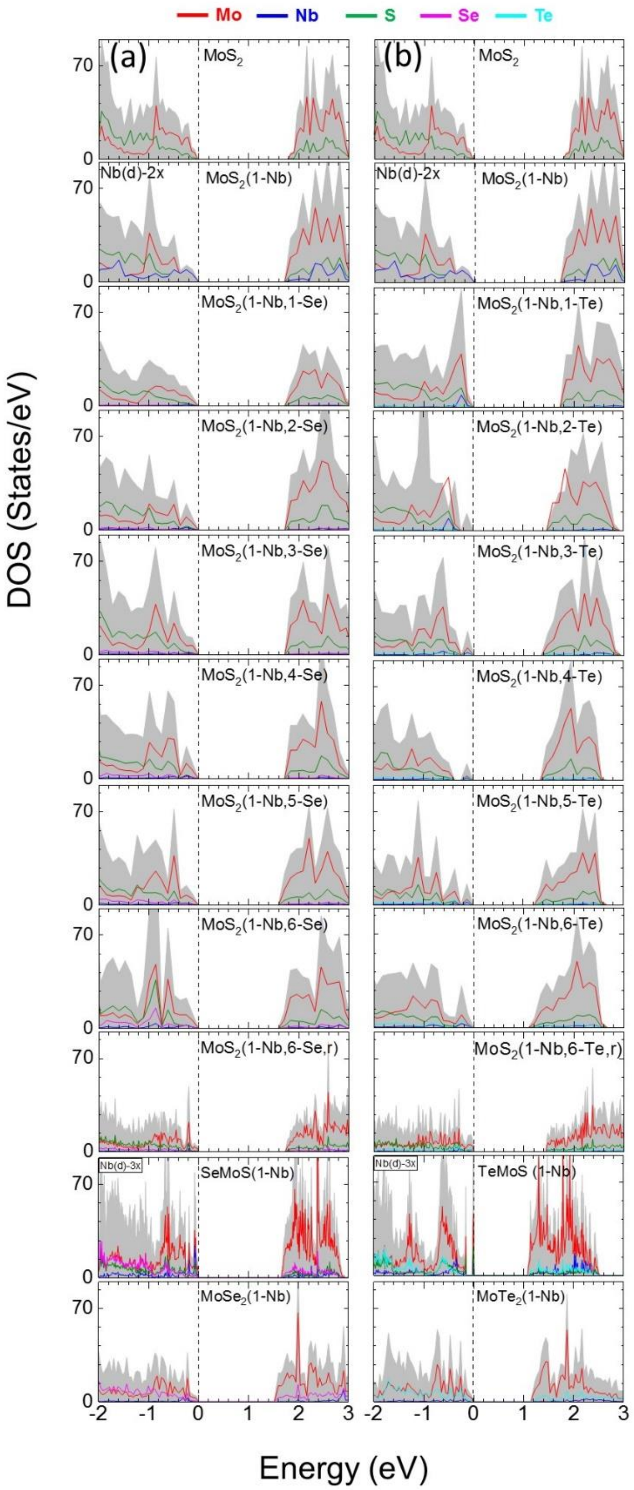

FIG. S2: Total and partial density of states of MoS_2, MoS_2(1-Nb), MoS_2(1-Nb,n-Se), SeMoS(1-Nb), $MoSe_2$(1-Nb) (left column) and MoS_2(1-Nb,n-Te), TeMoS(1-Nb), $MoTe_2$(1-Nb) (right column). Here n=1-6 and the valence band maximum was set to 0 eV.

SB. Formation energy

The formation or substitution energy of foreign atoms in monolayer (ML) MoS_2 is calculated under different chemical environmental conditions as described in Refs [1, 3] . The formation energy for nX substitutional atoms in the MoS_2 ML is calculated using

$$E_f = E[MoS_2(n\text{-}X)] - E_0 + n\mu_h - n\mu_X \qquad \text{(SA.1)}$$

where $E[MoS_2(n-X)]$ and E_0 are the total energy of the MoS_2(n-X) ML and the pristine MoS_2 ML, respectively. Note that n=1 if X=Nb, and n=1,…,6 if X=Se,Te. μ_h is chemical potential of the substituted atom of the host medium and μ_X is that of substitutional atom. Similarly, the formation energy for nY (Y=Se, Te) in MoS_2(1-Nb) is calculated from

$$E_f = E[MoS_2(1-Nb, n-Y)] - E[MoS_2(1-Nb)] + n\mu_h - n\mu_Y \qquad \text{(SA.2)}$$

$E[MoS_2(1-Nb, n-Y)]$ is the total energy of the MoS_2(1-Nb,n-Y) ML.

The chemical potential of Mo, Nb, S, Se, and Te is calculated using bulk Mo, Nb bcc crystals and S_2, Se_2, and Te_2 dimers as reference systems. The values of the chemical potential range from S-rich (or equivalently Mo-poor) to S-poor (or equivalently Mo-rich) conditions. The formation energy of the MoS_2 ML is determined using

$$E_f^{MoS_2} = \mu_{MoS_2} - \mu_{Mo}^0 - 2\mu_S^0 \quad \text{(SA.3)}$$

where μ_{Mo}^0 and μ_S^0 are total energy per atom of Mo and S reference systems. μ_{MoS_2} is total energy per formula unit of the MoS_2 ML. Under S-rich condition, the chemical potential of S is equal to μ_S^0 and chemical potential of Mo is defined as

$$\mu_{Mo}^{S-rich} = \mu_{Mo}^0 + E_f^{MoS_2} \quad \text{(SA.4)}$$

Under S-poor condition, chemical potential of Mo is equal to μ_{Mo}^0 and chemical potential of S is defined as

$$\mu_S^{S-poor} = \mu_S^0 + \frac{1}{2} E_f^{MoS_2} \quad \text{(SA.5)}$$

In this work the corresponding formation energies are determined under both the S-rich and S-poor conditions. The results are summarized in Tables S1, S2 and S3. Note that in the case of Janus layers the energy required for the substitution of a whole S layer by a Se or Te layer is determined similar to Eq. SA.1, and for a Janus layer with a Nb substitutional atom a relation similar to Eq. SA.2 is applied.

Table S1: Formation energy (E_f in eV per substitutional atom) of MoS_2(n-X) (n=1 - 6; X = Se, Te) and Janus MLs under S rich and Mo rich conditions.

	**S rich**	**Mo rich**	**S rich**	**Mo rich**
	**X= Se**		**X = Te**	
MoS_2(1-X)	0.249	-2.107	0.802	-1.554
MoS_2(2-X)	0.255	-2.101	0.832	-1.524
MoS_2(3-X)	0.261	-2.095	0.856	-1.500
MoS_2(4-X)	0.262	-2.094	0.860	-1.496
MoS_2(5-X)	0.266	-2.090	0.878	-1.478
MoS_2(6-X)	0.271	-2.086	0.899	-1.457
MoS_2(6-X,r)	0.258	-2.099	0.839	-1.517
XMoS	0.314	-2.042	0.956	-1.400

Table S2: Formation energy (E_f in eV per substitutional atom) of MoS_2(1-Nb,n-X) (n=1 - 6; x = Se, Te) and Janus MLs under S rich and Mo rich conditions

	**S rich**	**Mo rich**	**S rich**	**Mo rich**
MoS_2(1-Nb)	-4.720	-0.006		
	**X = Se**		**X = Te**	
MoS_2(1-Nb,1-X)	0.256	-2.100	0.815	-1.541
MoS_2(1-Nb,2-X)	0.264	-2.093	0.828	-1.528
MoS_2(1-Nb,3-X)	0.265	-2.092	0.833	-1.523
MoS_2(1-Nb,4-X)	0.269	-2.087	0.848	-1.509
MoS_2(1-Nb,5-X)	0.271	-2.085	0.873	-1.483
MoS_2(1-Nb,6-X)	0.275	-2.081	0.902	-1.454
MoS_2(1-Nb,6-X,r)	0.261	-2.097	0.833	-1.524
XMoS(1-Nb)	0.275	-2.082	0.927	-1.429

SC. Thermoelectric property: Seebeck coefficient at 500 and 800 K

The Seebeck coefficient(S) as function of Fermi energy at 500 and 800 K is presented in Fig S4 and S5, respectively. As mentioned in main text Janus layers have a smaller peak value than MLs with low Se/Te concentration. The S peak values are diminishing as T increases from 500 to 800 K.

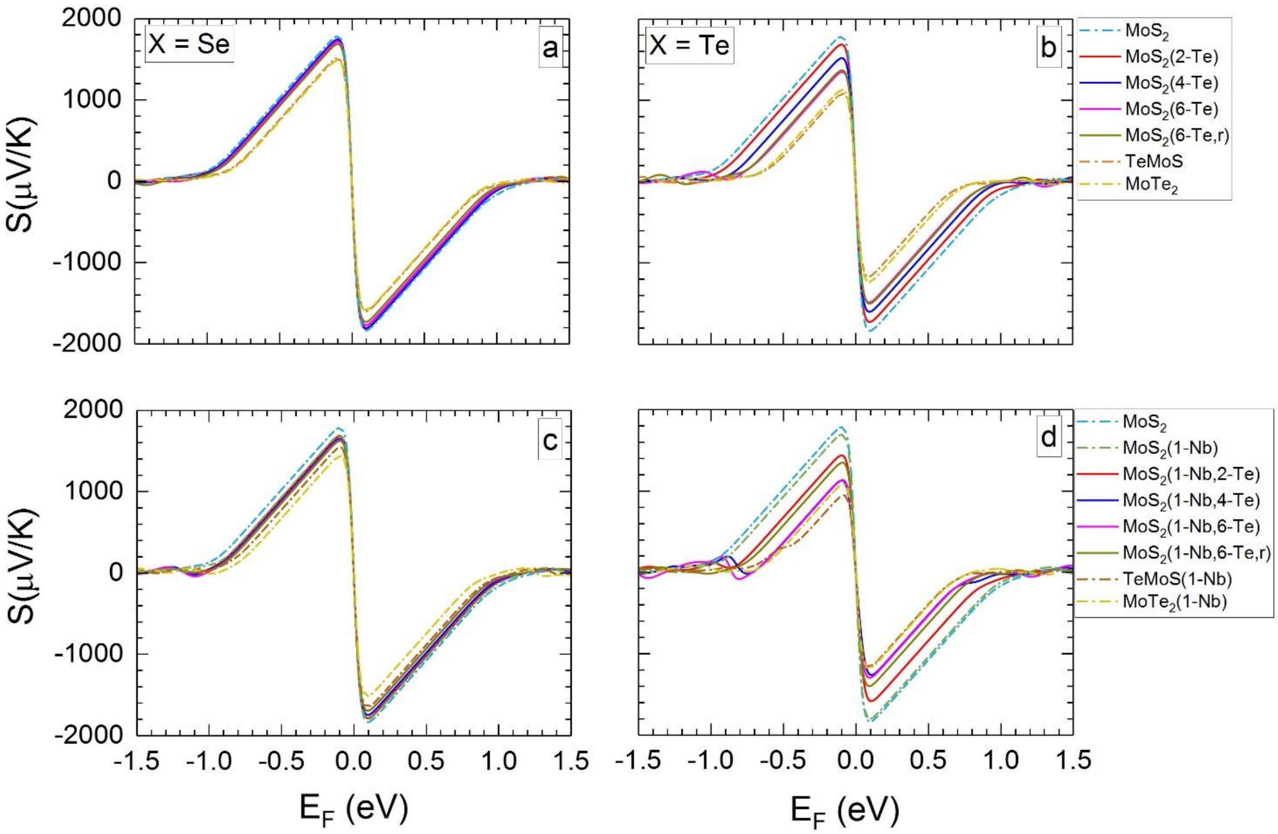

FIG. S4: Seebeck coefficients calculated for a) MoS_2(n-Se), b) MoS_2(n-Te), c) MoS_2(1-Nb,n-Se), and d) MoS_2(1-Nb,n-Te) MLs as a function of Fermi energy at temperature 500 K. The Seebeck coefficients of Janus layers SeMoS (a), TeMoS (b) , SeMoS(1-Nb) (c) , TeMoS(1-Nb), and of $MoSe_2$ (a), $MoTe_2$ (b), $MoSe_2$(1-Nb), as well as $MoTe_2$(1-Nb) MLs are also shown. n=2,4, and 6.

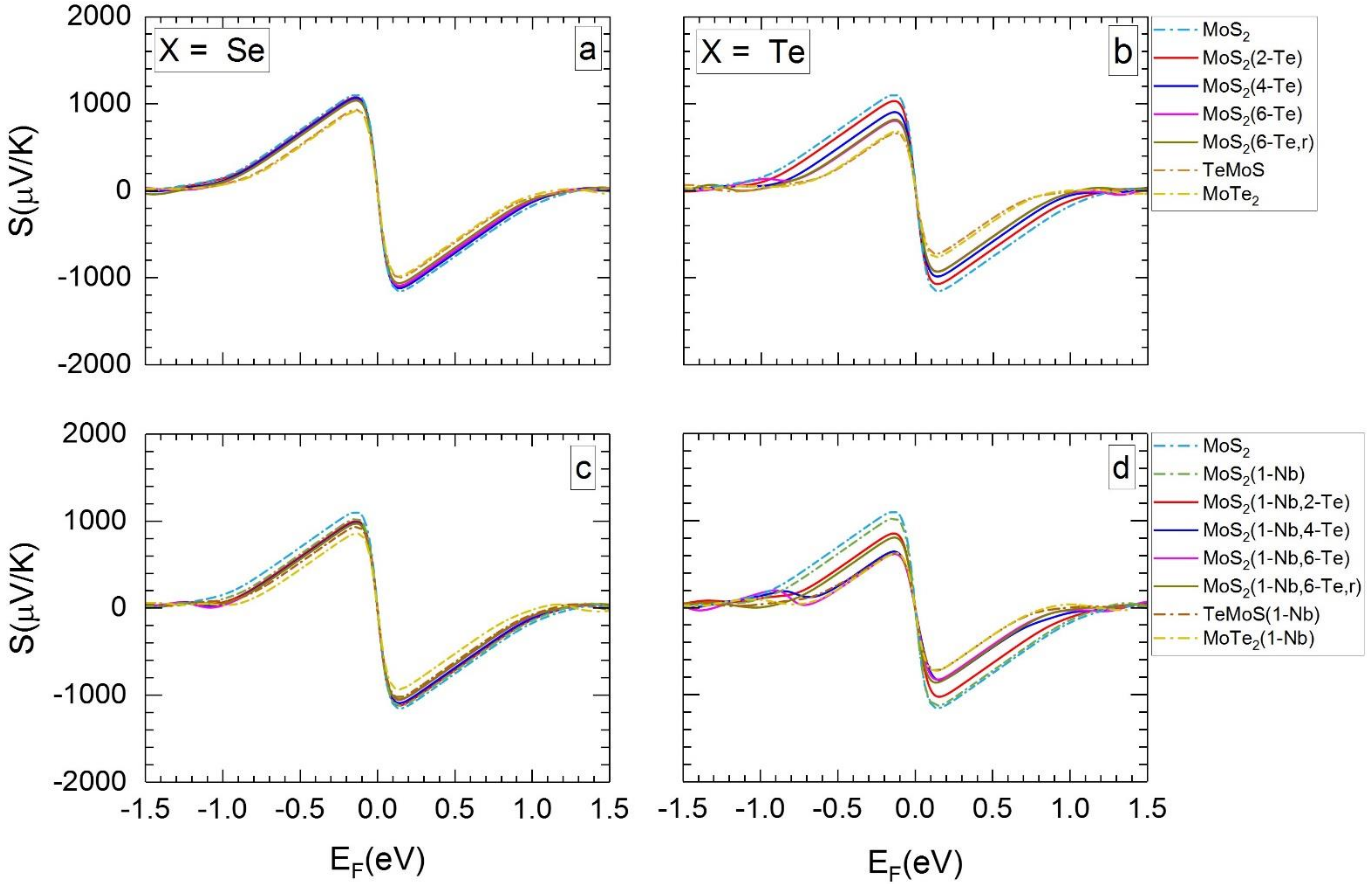

FIG. S5: Seebeck coefficients at temperature 800 K. For further details see Fig. S4.

SD. Thermoelectric property: TE figure of merit at 500 and 800 K

The thermoelectric figure of merit (ZT) as a function of Fermi energy at 500 and 800 K is presented in Fig S6 and S7 respectively. The maximum ZT of MLs with low Se/Te concentration is higher than that of Janus layers and MoX_2 MLs(X=S,Se,Te).

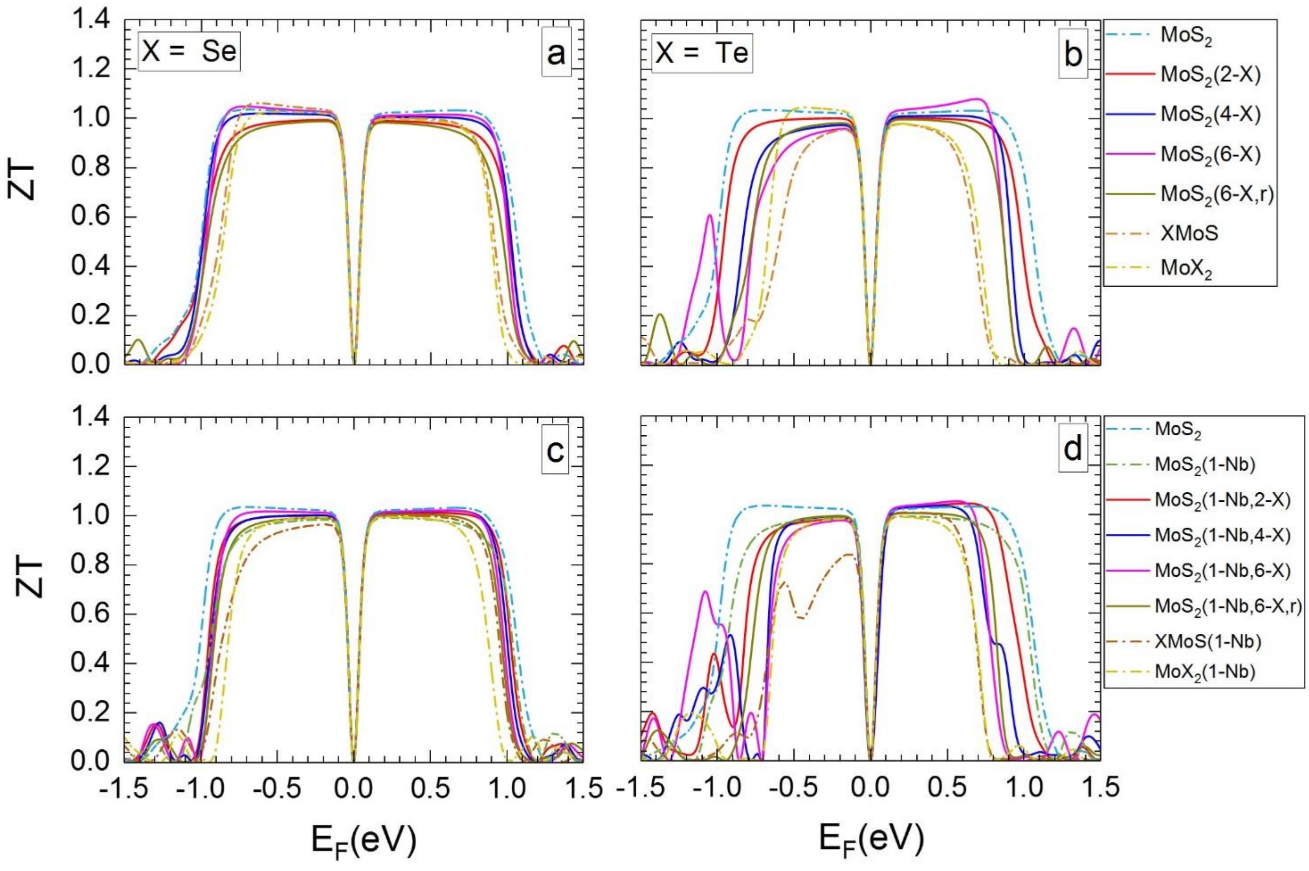

FIG. S6: TE figure of merit of a) MoS_2(n-Se), b) MoS_2(n-Te), c) MoS_2(1-Nb,n-Se), and d) MoS_2(1-Nb,n-Te) as a function of Fermi energy at temperature 500 K, along with that of Janus layers (a) SeMoS, (b) TeMoS, (c) SeMoS(1-Nb), and (d) TeMoS(1-Nb) as well as $MoSe_2$, $MoTe_2$, $MoSe_2$(1-Nb), and $MoTe_2$(1-Nb) MLs. n=2,4, and 6.

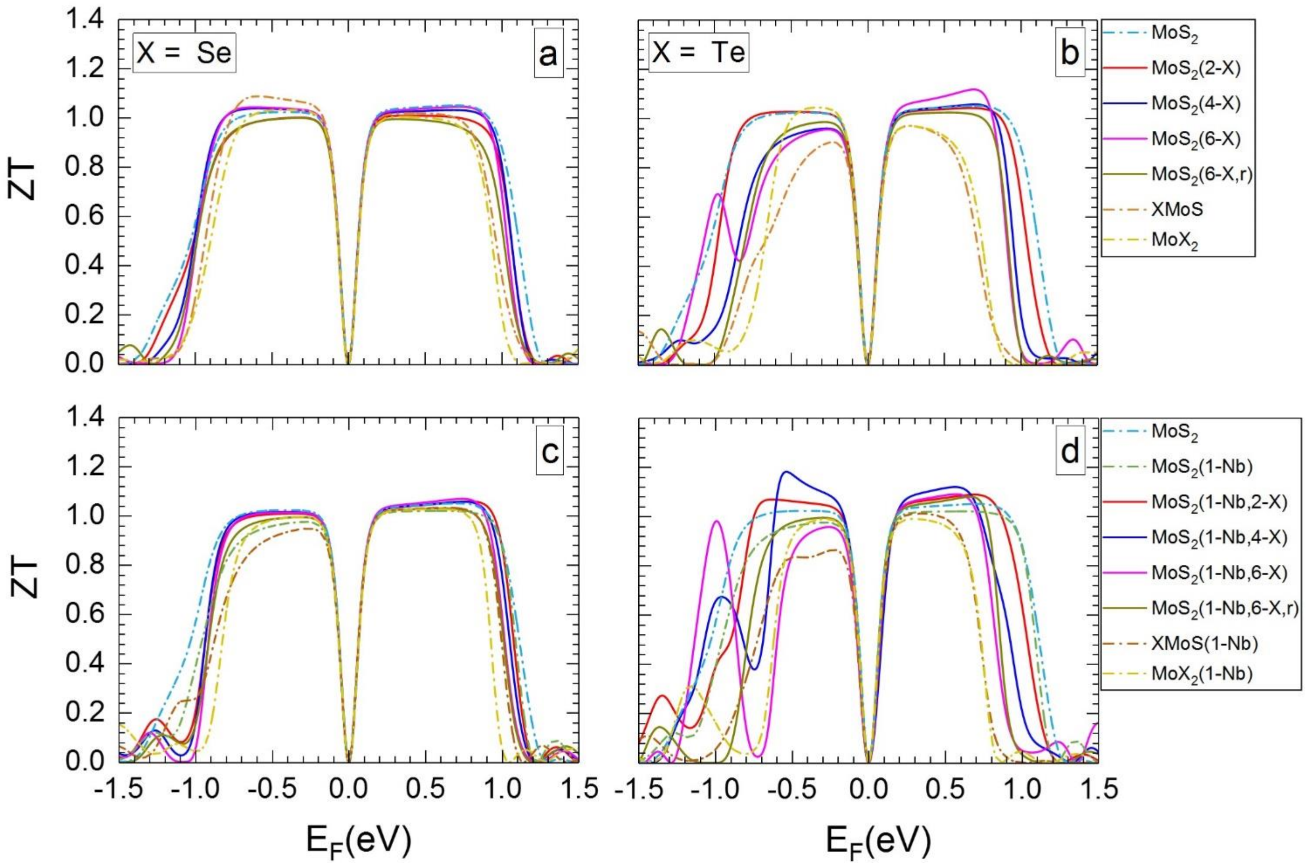

FIG. S7: TE figure of merit at temperature 800 K. For further details see Fig. S6.

SE. Band structure and thermal properties of TeMoS and TeMoS(1-Nb) under different lattice parameter

We have selected TeMoS and TeMoS(1-Nb) Janus layers to test their band structure and thermoelectric properties under different lattice parameter. We have increased the lattice parameter by 7%. The calculated band structure, Seebeck coefficient and Figure of merit are shown in Fig. S8, Fig. S9 and Fig. S10 respectively. We observed neligible changes in band structure profile and bandgap values.

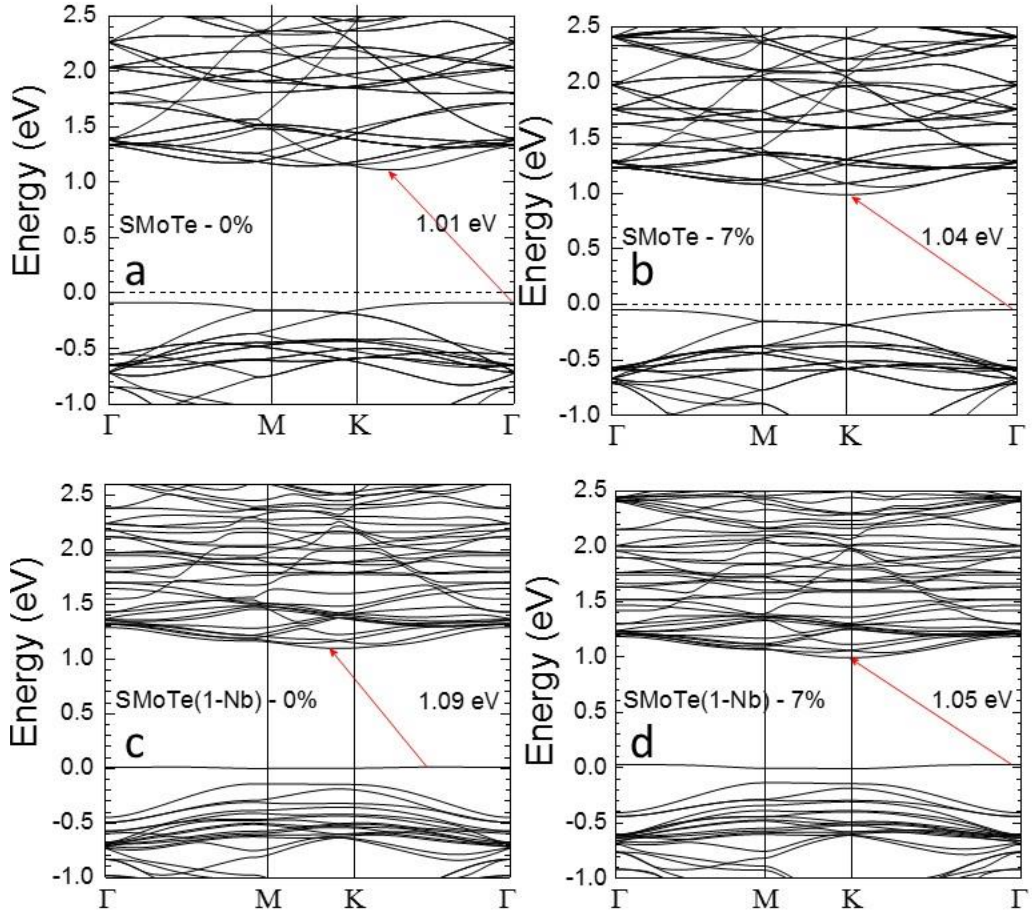

FIG. S8: The band structure plots for a) TeMoS without increase in lattice parameter, b) TeMoS with 7% increase in lattice parameter, c) TeMoS(1-Nb) without increase in lattice parameter, and d) TeMoS(1-Nb) with 7% increase in lattice parameter.

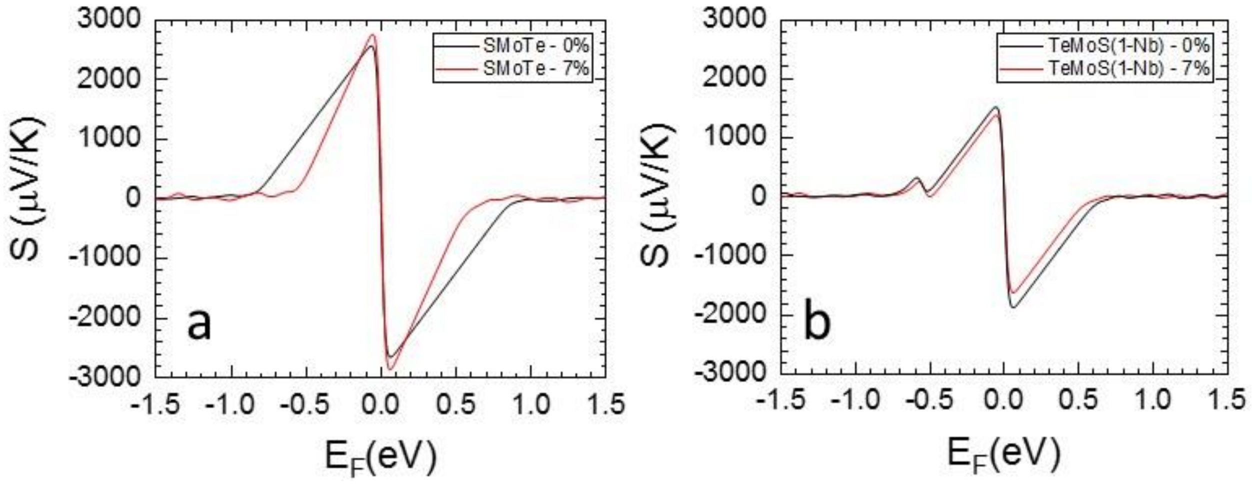

FIG. S9: The calculated Seebeck coefficient for a) TeMoS with and without increase in lattice parameter, b) TeMoS(1-Nb) with and without increase in lattice parameter.

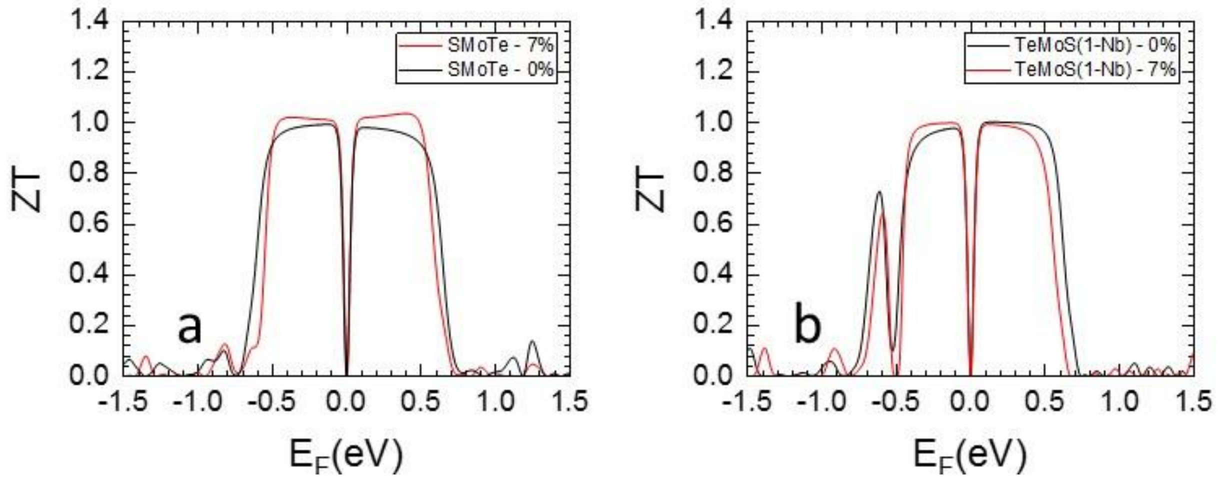

FIG. S10: The calculated Figure of merit for a) TeMoS with and without increase in lattice parameter, b) TeMoS(1-Nb) with and without increase in lattice parameter.

SF. Supplement References

1. K. Dolui, I. Rungger, C. Das Pemmaraju and S. Sanvito, *Phys. Rev. B*, 2013, **88**, 075420. https://doi.org/10.1103/PhysRevB.88.075420

2. K. Ikeura, H. Sakai, M. S. Bahramy and S. Ishiwata, *APL Mater.*, 2015, **3**, 041514. https://doi.org/10.1063/1.4913967

3. H. Wan, L. Xu, W.-Q. Huang, J.-H. Zhou, C.-N. He, X. Li, G.-F. Huang, P. Peng and Z.-G. Zhou, *RSC Adv.*, 2015, **5**, 7944. https://doi.org/10.1039/c4ra12498g
